# Faecal haemoglobin concentration thresholds for reassurance and urgent investigation for colorectal cancer based on a faecal immunochemical test in symptomatic patients in primary care

**DOI:** 10.1177/0004563220985547

**Published:** 2021-01-21

**Authors:** Craig Mowat, Jayne Digby, Judith A Strachan, Rebecca K McCann, Francis A Carey, Callum G Fraser, Robert JC Steele

**Affiliations:** 1 Department of Gastroenterology, University of Dundee, School of Medicine Ninewells Hospital and Medical School, Dundee, Scotland, UK; 2 Centre for Research into Cancer Prevention and Screening, University of Dundee, School of Medicine, Ninewells Hospital and Medical School, Dundee, Scotland, UK; 3 Department of Blood Sciences, Ninewells Hospital and Medical School, NHS Tayside, Dundee, Scotland, UK; 4 Department of Pathology, Ninewells Hospital and Medical School, NHS Tayside, Dundee, Scotland, UK

**Keywords:** Colorectal cancer, colorectal disease, faecal biomarkers, faecal immunochemical test, faecal haemoglobin, primary care

## Abstract

**Background:**

Faecal haemoglobin concentration (f-Hb), estimated using a faecal immunochemical test, can be safely implemented in primary care to assess risk of colorectal cancer (CRC). Clinical outcomes of patients presenting with symptoms of lower gastrointestinal disease were examined using an extensive range of f-Hb thresholds to decide on reassurance or referral for further investigation.

**Methods:**

All patients who attended primary care and submitted a single faecal specimen faecal immunochemical test in the first year of the routine service had f-Hb estimated using HM-JACKarc: f-Hb thresholds from <2 to ≥ 400 µg Hb/g faeces (µg/g) were examined.

**Results:**

Low f-Hb thresholds of <2, <7, <10 and <20 µg/g gave respective CRC risks of 0.1, 0.3, 0.3 and 0.4%, numbers needed to scope for one CRC of 871, 335, 300 and 249, and ‘false negative’ rates of 2.9, 11.4, 13.3 and 17.1%. With thresholds of <2, <7, <10 and <20 µg/g, 48.6, 74.6, 78.1 and 83.2% respectively of symptomatic patients could be managed without further investigation. With reassurance thresholds of <2 µg/g, <7 µg/g and <10 µg/g, the thresholds for referral for urgent investigation would be >400 µg/g, ≥200 µg/g and ≥100 µg/g. However, patients with a f-Hb concentration of <10 or <20 µg/g with iron deficiency anaemia, or with severe or persistent symptoms, should not be denied further investigation.

**Conclusions:**

In primary care, f-Hb, in conjunction with clinical assessment, can safely and objectively determine individual risk of CRC and decide on simple reassurance or urgent, or routine referral.

## Introduction

Lower gastrointestinal (GI) symptoms are poor predictors of colorectal cancer (CRC), and other serious bowel disease.^1^ When guidance on the ‘two week wait’ for urgent referral for further investigation of patients with symptoms suspicious of CRC was introduced in England, this led to a large increase in referrals, but no change in the stage of diagnosis of CRC.^2^ In addition, in a recent study, patients from primary care practices with the highest urgent suspected cancer referral rates did not have lower likelihood of late stage diagnosis than those from practices with lower referral rates.^3^

The problem with relying on symptoms alone is that all potentially caused by CRC are often due to non-significant or functional disorders.^1^ This is compounded by the fact that current guidance, both from Scottish Government^4^ and the National Institute for Health and Care Excellence (NICE) in England,^5,6^ has serious limitations, since it is mainly based on the presenting symptoms and, in the latter, very dependent on age. In addition, NICE are currently engaged in considering development of guidance on quantitative faecal immunochemical tests (FIT) to guide CRC referral for patients presenting in primary care with a change in bowel habit or abdominal pain.^7^

The available current guidance in the UK is complex and open to wide interpretation by general practitioners (GP). However, there is burgeoning evidence that using faecal haemoglobin concentration (f-Hb), as estimated by quantitative FIT, allows a rational, universal approach to identifying those symptomatic patients who would benefit most from further investigation^8^ and those who can be safely managed with what we think is appropriately termed a ‘reassurance’ strategy. This strategy involves giving advice to patients that their symptoms are unlikely to be due to significant GI disease, but they should seek advice should their symptoms return, continue, or worsen. Further, it is now clear that FIT can be employed in this context for all symptomatic patients, not only low-risk^9,10^ as recommended in NICE NG12^6^ and DG30,^7^ but also those who report high-risk symptoms,^11–15^ particularly rectal bleeding.^16^

However, uncertainty remains around a numerical f-Hb threshold to discriminate between those who are and are not likely to benefit from investigation. There is even less consensus on the f-Hb threshold that should trigger urgent investigation. For example, in response to the severe constraints on colonoscopy delivery imposed by the current COVID-19 pandemic, National Health Service (NHS) England has recommended a threshold of 100 µg/g for urgent investigation^17^ whereas similar guidance from NHS Scotland recommends 400 µg/g.^18^ In an ideal world, there would be no need to distinguish between urgent and routine referrals but this is currently unrealistic in the United Kingdom (UK) and probably in many other countries. The need for a rational approach to determining which patients with symptoms should be further investigated urgently will continue for the foreseeable future.^19^

Therefore, when using f-Hb as an aid to categorizing patients with lower GI symptoms, it is crucial to be able to decide on thresholds for reassurance and urgent referral based on robust data that encompass the complete range of possible f-Hb thresholds. In addition, for FIT to have maximum benefit in terms of efficient utilization of services and reassurance of patients, it is essential, in our view, that it is employed in primary care and is requested, with few exceptions, on every patient who presents with lower GI symptoms.^20,21^

For these reasons, the CRC diagnostic outcomes across a comprehensive range of f-Hb thresholds in a wide spectrum of patients who presented with lower GI symptoms and had FIT in primary care were studied. These data were collected over a one-year period in a region in which GPs have been encouraged since 2015 to use FIT in such patients regardless of the specific symptoms, and to use f-Hb <10 µg/g as an indication for reassurance without referral for further investigation.^20^ The introduction of this service and the performance of a 10 µg/g f-Hb threshold has been described previously.^14^

## Methods

In the NHS Tayside Board, FIT kits (Hitachi Chemical Diagnostics Systems Co., Ltd, Tokyo, Japan, supplied by Alpha Labs Ltd, Eastleigh, Hants, UK) were made available to GP practices beginning in December 2015. GPs were recommended to request f-Hb to guide referral of patients with any lower GI symptoms, along with a full blood count. Patients were requested to return the completed FIT specimen collection device immediately to the GP facility and, from there, the devices were delivered to Blood Sciences, Ninewells Hospital and Medical School, Dundee, at ambient temperature, by the routine sample collection service and, if required, stored at 4 °C prior to analysis. Analyses were carried out from Monday through Friday; most samples were analysed on the day of receipt and results reported electronically to the requesting GP after f-Hb measurement using one HM-JACKarc (Hitachi Chemical Diagnostics Systems) FIT system which has a limit of detection (LoD) of 2 μg/g, a limit of quantitation (LoQ) of 7 μg/g and an upper measurement limit of 400 µg/g.^22^ Samples with results above the upper measurement limit were therefore reported as >400 μg/g. In our routine practice, using a single threshold, as previously described in detail,^14^ patients with f-Hb ≥ 10 µg/g were defined as worthy of further investigation as recommended in NICE DG30.^6^ The reports also sign-posted GPs to web-based advice that f-Hb <10 µg/g, in the absence of iron deficiency anaemia (IDA), severe persistent symptoms, or a rectal or abdominal mass, suggests that CRC is extremely unlikely.

Numerical FIT results generated from 7 December 2015 to 7 December 2016 were retrieved from the laboratory database and linked, using the Community Health Index (CHI) number, with the electronic patient record to access all correspondence, laboratory results, referrals to secondary care, colonoscopy findings, hospital admissions and attendances at the primary care out-of-hours service. In addition, in December 2018, the Health Informatics Centre, University of Dundee, used the CHI number of all patients who had submitted a FIT to the laboratory to perform a *post-hoc* anonymized record linkage with the Scottish Cancer Registry (SCR). This was carried out in order to identify any cases of CRC that had been overlooked (International Classification of Diseases [ICD] codes C18, C19 and C20). All cases of CRC were confirmed histologically. MedCalc statistical software (MedCalc Software, Mariakerke, Belgium) was employed for calculations.

## Results

The data presented here include all patients in the study period who presented to their GP and had a f-Hb result, irrespective of whether they had been referred or investigated further. The results are given in Tables 1 [Table table2-0004563220985547][Table table3-0004563220985547]to 4.

**Table 1.** Test performance of FIT below different faecal haemoglobin concentration (f-Hb) thresholds (µg Hb/g faeces) for colorectal cancer (CRC) in all patients with f-Hb.

f-Hb threshold (µg Hb/g faeces)	n	Percentage below threshold	CRC absent	CRC present	CRC not referred initially for investigation	Specificity (95%CI)	Negative predictive value (95% CI)	False negative proportion (% CRC missed) (95% CI)	CRC risk amongst those below threshold (%)	Number needed to investigate to find one cancer
<2	2614	48.6%	2611	3	2	49.5% (48.1–50.9)	99.9% (99.7–100)	2.9% (0.6–8.1)	0.1%	871
<7	4016	74.6%	4004	12	6	75.9% (74.7–77.0)	99.7% (99.5–99.8)	11.4% (6.1–19.1)	0.3%	335
<10	4204	78.1%	4190	14	6	79.4% (78.3–80.5)	99.7% (99.5–99.8)	13.3% (7.5–21.4)	0.3%	300
<20	4477	83.2%	4459	18	6	84.5% (83.5–85.5)	99.6% (99.4–99.7)	17.1% (10.5–25.7)	0.4%	249
<50	4733	88.0%	4706	27	7	89.2% (88.3–90.0)	99.4% (99.2–99.6)	25.7% (17.7–35.2)	0.6%	175
<100	4878	90.7%	4846	32	8	91.8% (91.1–92.6)	99.3% (99.1–99.5)	30.5% (21.9–40.2)	0.7%	152
<150	4962	92.2%	4924	38	8	93.3% (92.6–94.0)	99.2% (99.0–99.4)	36.2% (27.0–46.2)	0.8%	131
<200	5003	93.0%	4963	40	9	94.1% (93.4–94.7)	99.2% (99.0–99.4)	38.1% (28.8–48.1)	0.8%	125
<250	5033	93.5%	4990	43	9	94.6% (93.9–95.2)	99.1% (98.9–99.3)	41.0% (31.5–51.0)	0.9%	117
<300	5061	94.1%	5015	46	9	95.1% (94.4–95.6)	99.1% (98.9–99.3)	43.8% (34.1–53.8)	0.9%	110
<350	5077	94.4%	5029	48	9	95.3% (94.7–95.9)	99.1% (98.8–99.2)	45.7% (36.0–55.7)	1.0%	106
<400	5089	94.6%	5040	49	9	95.5% (94.9–96.1)	99.0% (98.8–99.2)	46.7% (36.9–56.7)	1.0%	103

**Table 4.** Characteristics of all the patients with faecal haemoglobin concentration (f-Hb) <20µg Hb/g faeces who were diagnosed with CRC, either as a result of their initial referral immediately after the result or subsequently identified by interrogating the available databases or linkage to the Scottish Cancer Registry.

f-Hb (µg Hb/g faeces	Age (years)	Sex	Symptoms	Initially referred	Blood Hb (g/l)	Tumour Size (mm)	Tumour Site	Dukes’ Stage
0	65	M	Pelvic pain, weight loss	No	126^a^	?	Rectum	?
1	78	M	Change of bowel habit	No	120^a^	26	Ascending Colon	A
1	83	F	Weight loss, change of bowel habit, rectal bleeding		103^a^	?	Caecum	D
2	61	F	Alternating diarrhoea/constipation	No	139	?	Transverse	D
2.5	89	M	Diarrhoea	Yes	71^a^	17	Transverse	A
3	74	F	Diarrhoea	Yes	102^a^	60	Caecum	?
3	87	F	Fatigue	Yes	108^a^	28	Caecum	A
3	67	M	Change of bowel habit, weight loss	No	162	32	Transverse Colon	D
5	84	F	Weight loss	No	98^a^	?	Caecum	D
5	70	M	Abdo Pain	No	151	53	Splenic Flexure	C
6	76	M	Change of bowel habit	Yes	138	?	Caecum	?
7	54	F	Diarrhoea	Yes	150	30	Rectum	A
7	58	M	Rectal bleeding	Yes	134	?	?	?
10	66	M	Change of bowel habit	Yes	94^a^	57	Transverse Colon	B
10	50	F	Change of bowel habit	Yes	154	67	Caecum	B
11	77	F	Diarrhoea	Yes	150	15	Rectum	A
12	80	M	Rectal Bleeding	Yes	123^a^	32	Sigmoid Colon	C
18	82	F	Fatigue	Yes	103^a^	40	Caecum	B

^a^Anaemia.

Table 1 shows the performance of FIT in patients with symptoms at a range of f-Hb thresholds that could be used to define a f-Hb that made a diagnosis of CRC very unlikely (i.e., the f-Hb that the GP could use to provide reassurance to the patient that significant GI disease was absent): note that the sixth column gives the number of CRC that were not initially referred in response to the f-Hb.

Table 2 shows the performance at a range of f-Hb thresholds that could be used to define a result that could make the diagnosis of CRC more likely (i.e., the results that the GP could use to justify referral for further investigation).

**Table 2. table2-0004563220985547:** Test performance of FIT above different faecal haemoglobin concentration (f-Hb) thresholds (µg Hb/g faeces for colorectal cancer (CRC) in all patients with f-Hb.

f-Hb threshold (µg Hb/g faeces)	n	Percentage above threshold	CRC absent	CRC present	Sensitivity (%)	False positive proportion (%)	Positive predictive value (%)	Number needed to investigate to find one cancer
≥2	2767	51.4%	2665	102	97.1% (91.9–99.4)	96.3% (95.5–97.0)	3.7% (3.5–3.8)	27
≥7	1365	25.4%	1272	93	88.6% (80.9–94.0)	93.2% (91.7–94.4)	6.8% (6.3–7.4)	15
≥10	1177	21.9%	1086	91	86.7% (78.6–92.5)	92.3% (90.6–93.7)	7.7% (7.1–8.4)	13
≥20	904	16.8%	817	87	82.9% (74.3–89.5)	90.4% (88.2–92.2)	9.6% (8.7–10.6)	10
≥50	648	12.0%	570	78	74.3% (64.8–82.3)	88.0% (85.1–90.3)	12.0% (10.7–13.6)	8
≥100	503	9.3%	430	73	69.5% (59.8–78.1)	85.5% (82.0–88.4)	14.5% (12.7–16.6)	7
≥150	419	7.8%	352	67	63.8% (53.9–73.0)	84.0% (80.1–87.3)	16.0% (13.8–18.5)	6
≥200	378	7.0%	313	65	61.9% (51.9–71.2)	82.8% (78.5–86.4)	17.2% (14.7–20.0)	6
≥250	348	6.5%	286	62	59.0% (49.0–68.6)	82.2% (77.7–86.0)	17.8% (15.1–20.9)	6
≥300	320	5.9%	261	59	56.2% (46.2–65.9)	81.6% (76.8–85.6)	18.4% (15.5–21.7)	5
≥350	304	5.6%	247	57	54.3% (44.3–64.0)	81.3% (76.3–85.4)	18.8% (15.7–22.2)	5
≥400	292	5.4%	236	56	53.3% (43.3–63.1)	80.8% (75.7–85.1)	19.2% (16.0–22.8)	5

Table 3 shows the risk of CRC associated with four f-Hb values that might be used to direct patients into the reassurance category (<2, <7, <10 and <20 µg/g) and the full range of f-Hb thresholds up to ≥400 µg/g that might stimulate referral for further investigation, along with the proportion of all patients represented. The lower f-Hb values were chosen because 2 µg/g is the LoD, 7 µg/g is the LoQ, <10 µg/g was used as the value that was communicated to the GP as a robust indication that, with the exceptions noted earlier, further investigation was not generally required, as per NICE DG30^6^ and <20 µg/g has been recommended as the most appropriate f-Hb value for the rather different clinical setting of asymptomatic population screening for CRC^23^ and has been used in assessment of symptomatic patients in at least one other study.^24^

**Table 3. table3-0004563220985547:** Risk of CRC associated with the intermediate faecal haemoglobin concentrations (f-Hb) between the four lowest ‘negative’ thresholds (<2, <7, <10 and <20 µg Hb/g faeces) and a range of thresholds for further investigation up to ≥400 µg Hb/g faeces along with the proportion of all patients.

f-Hb (µg Hb/g faeces)	Risk of CRC (%)	Proportion of all patients (%)	FHbf-Hb (µg Hb/g faeces)	Risk of CRC (%)	Proportion of all patients (%)	f-Hb (µg Hb/g faeces)	Risk of CRC (%)	Proportion of all patients (%)	f-Hb (µg Hb/g faeces)	Risk of CRC (%)	Proportion of all patients (%)
<2	0.1	48.6	<7	0.3	74.6	<10	0.3	78.1	<20	0.4	83.2
2–399	1.9	46.0	7–399	3.4	19.9	10–399	4.0	16.4	20–399	5.1	11.4
>400	19.2	5.4	>400	19.2	5.4	>400	19.2	5.4	>400	19.2	5.4
2–349	1.8	54.8	7–349	3.4	19.7	10–349	3.9	16.2	20–349	5.0	11.2
>350	18.8	5.6	>350	18.8	5.6	>350	18.8	5.6	>350	18.8	5.6
2–299	1.8	45.5	7–299	3.3	19.4	10–299	3.7	15.9	20–299	4.8	10.9
>300	18.4	5.9	>300	18.4	5.9	>300	18.4	5.9	>300	18.4	5.9
2–249	1.7	45.0	7–249	3.0	18.9	10–249	3.5	15.4	20–249	4.5	10.3
>250	17.8	6.5	>250	17.8	6.5	>250	17.8	6.5	>250	17.8	6.5
2–199	1.5	44.4	7–199	2.8	18.3	10–199	3.3	14.8	20–199	4.2	9.8
>200	17.2	7.0	>200	17.2	7.0	>200	17.2	7.0	>200	17.2	7.0
2–149	1.5	43.6	7–149	2.7	17.6	10–149	3.2	14.1	20–149	4.1	9.0
>150	16.0	7.8	>150	16.0	7.8	>150	16.0	7.8	>150	16.0	7.8
2–99	1.3	42.1	7–99	2.3	16.0	10–99	2.7	12.5	20–99	3.5	7.4
>100	14.5	9.3	>100	14.5	9.3	>100	14.5	9.3	>100	14.5	9.3
2–49	1.1	39.4	7–49	2.1	13.3	10–49	2.5	9.8	20–49	3.5	4.8
>50	12.0	12.0	>50	12.0	12.0	>50	12.0	12.0	>50	12.0	12.0
2–19	0.8	34.6	7–19	1.3	8.6	10–19	1.5	5.1	>20	9.6	16.8
>20	9.6	16.8	>20	9.6	16.8	>20	9.6	16.8			
2–9	0.7	29.5	7–9	1.1	3.5	>10	7.7	21.9			
>10	7.7	21.9	>10	7.7	21.9						
2–7	0.6	26.1	>7	6.8	25.4						
>7	6.8	25.4									
>2	3.7	51.4									

Table 4 shows the characteristics of all the patients with a f-Hb <20 µg/g who were diagnosed with CRC, either as a result of their initial referral immediately after the f-Hb result, or subsequently identified by interrogating the available databases or linkage to the SCR.

## Discussion

In a previous publication,^14^ we described in considerable detail the implementation of a routine FIT service in primary care as an adjunct to diagnostic decision-making, using a threshold of <10 µg/g that would indicate that it was sufficiently unlikely to have CRC to warrant the approach we term ‘reassurance’. The use of FIT was not mandated, but the number of requests increased steadily, and, overall, 70.5% of referrals for investigation were accompanied by a request for FIT. Of the f-Hb recorded that were not associated with an immediate referral (n = 2521), 95.3% were <10 µg/g. However, the f-Hb was not used as a rigid criterion to exclude patients from investigation and, of those patients who were referred (n = 2848), 63.0% had a f-Hb <10 µg/g. The introduction of this service led to an overall reduction in referrals to secondary care of 15.1% in the first year, and of those not immediately referred, only six (0.2%) were subsequently diagnosed with CRC, with no evidence of adverse outcome from diagnostic delay.

The diagnostic outcomes associated with a wide range of f-Hb thresholds have been documented here to assist in making a rational decision as to which thresholds to use for advocating reassurance and for indicating the need for urgent or other further investigation.

A threshold of <10 µg/g is now widely used in assessment of patients with symptoms. The evidence supporting this is largely based on the first studies on FIT in the symptomatic done in Scotland which documented receiver operating characteristic analyses that gave the optimum balance between sensitivity and specificity for CRC detection as 10 µg/g,^25,26^ supported by a larger similar study from Spain.^27^ Others have used different thresholds.^21^ However, because some CRC do have f-Hb below the NICE DG30 recommended threshold,^6^ interest focused (before the COVID-19 pandemic) on use of lower f-Hb thresholds, approaching the LoD and LoQ of the FIT system used.^22^ These include undetectable,^20^ 2,^11^ 4,^12,28^ and 7 µg/g.^29,30^ CRC detection did improve at lower f-Hb thresholds with higher clinical sensitivity, but at the expense of higher positivity and colonoscopy demand, and lower positive predictive value (PPV).

In contrast, few have studied higher f-Hb thresholds but, because of the imperative to prioritize patients for further investigation in the current COVID-19 pandemic, a f-Hb threshold of 100 µg/g has been suggested for England despite the clinical characteristics being unknown at present.^17^ Others have proposed 150 µg/g for use as a threshold in assessment of patients with symptoms,^31,32^ the rationale being that this threshold gives positivity approximating to that found in the past in the NHS Bowel Cancer Screening Programme with guaiac faecal occult blood tests.^33^

However, the dataset provided here can be used to guide a rational choice of numerical f-Hb thresholds for use in primary care to guide the investigation of symptomatic patients. Table 1 shows that reassurance f-Hb thresholds of 2, 7, 10 and 20 µg/g give respective CRC risks of 0.1, 0.3, 0.3 and 0.4%, numbers needed to scope for one CRC of 871, 335, 300 and 249, and ‘false negative’ rates of 2.9, 11.4, 13.3 and 17.1%. When colonoscopy is employed as a first-line asymptomatic population screening test, it has been reported that investigation of 111 asymptomatic individuals between the ages of 50 and 66 years is needed to detect one CRC^34^; thus, the use of FIT in symptomatic patients is extremely effective at identifying those at lower risk than the asymptomatic population at large. In addition, since colonoscopy in England is associated with a 7.4% ‘false negative rate’ as assessed by the three-year post-colonoscopy CRC rate,^35^ a f-Hb threshold of 2 µg/g could be said to perform better than colonoscopy for reassurance that significant disease is absent.

It is important to stress, however, that, unlike colonoscopy, FIT is not a diagnostic test, but rather an aid to diagnosis and, in our initial experience, a f-Hb of <10 µg/g did not deter GPs from referring patients when a rational clinical indication existed. However, as GPs gain further confidence in FIT, it is likely that referral of patients with low f-Hb will become less common. The reassurance threshold chosen will have a direct effect on colonoscopy demand, and using thresholds of 2, 7, 10, or 20 µg/g would mean that 48.6, 74.6, 78.1 and 83.2% respectively of the symptomatic population could be managed without further investigation.

However, no matter what f-Hb reassurance threshold is used, some CRC will be missed, and it pays dividends to look at the characteristics of the patients found to have CRC, but with f-Hb <20 µg/g. This allows a logical approach to safety-netting for those patients with low f-Hb, and Table 4 shows that 9 of the 18 patients (50.0%) with f-Hb concentrations of <20 µg/g who were found to have CRC had anaemia (with an iron deficiency pattern). Therefore, a patient with a f-Hb concentration of <10 or <20 µg Hb/g faeces with iron deficiency anaemia (IDA), or with severe and persistent symptoms, should undergo further investigation. It is also important to recognize the clinical experience and expertise of GPs in assessing the nature and severity of symptoms: ‘gut feelings for cancer’ can be conceptualized as a rapid summing up of multiple verbal and non-verbal patient cues.^36^ Thus, f-Hb should not be used by secondary care to refuse requests from GPs to have a patient investigated, but rather it should be used by the GPs themselves to assist in reaching an objective decision as to whether or not to refer for further investigation.

Unsurprisingly, the focus of research into FIT for symptomatic patients has been on the ‘reassurance’ f-Hb threshold as this affects demand for bowel investigation. It would be ideal to be able to offer prompt investigation to everyone with f-Hb higher than the reassurance threshold but, with current constraints, determining thresholds to distinguish between patients requiring no, routine or urgent, investigation is necessary for efficient and effective patient care. Germane to deciding an appropriate threshold in this context is the decision taken by the NICE Guideline Development Group responsible for the generation of NG12^5^; it was agreed to use a 3% PPV threshold value to underpin the recommendations for urgent investigation for suspected cancer. If the data presented here are treated as dichotomous, then all symptomatic patients with a f-Hb <400 µg/g had a PPV for CRC of under 3% (Table 1) but if a subset of patients with a low f-Hb (<2, <7, <10 or <20 µg/g) are to be reassured and not investigated, this markedly changes the significance of higher thresholds (Table 2).

As shown in Table 3, the risk of CRC when the f-Hb is in the intermediate range between the reassurance and urgent investigation thresholds varies owing to changes in the numbers of patients embraced within the intermediate range. Thus, with a reassurance f-Hb threshold of <2 µg/g, the risk does not approach 3% in any of the intermediate ranges, so that a f-Hb threshold of ≥400 µg/g would seem appropriate for urgent investigation. However, with a reassurance threshold of <7 µg/g, a CRC risk of 3% is reached at the 7–249 µg/g f-Hb range, so that a threshold for urgent referral of ≥200 µg/g could be employed, and, using the same logic, with a reassurance threshold of <10 µg/g, the threshold for urgent referral would be ≥100 µg/g. Were a reassurance threshold of <20 µg/g to be employed, no intermediate range PPV falls below 3%, so that every patient with a f-Hb ≥20 µg/g would be classified as requiring urgent investigation, but this group would only make up 16.8% of patients presenting with symptoms.

Interestingly, an economic evaluation carried out for the UK National Screening Committee estimated that the most cost-effective approach to CRC screening is biennial FIT at a threshold of 20 µg/g in the 50–74 age range,^23^ which would give a positivity rate of around 8% in participants.^33^ Thus, if a reassurance threshold of <20 µg/g was adopted for symptomatic patients, which would give a positivity rate of 16.8%, the same threshold could be adopted for participants in screening and for symptomatic patients. This would have the virtue of simplicity and, if the reassurance threshold was adhered to for patients with symptoms, with the proviso of adequate safety-netting, the reduction in demand for symptomatic bowel investigation could be sufficient to accommodate much lower screening f-Hb thresholds than those currently used in the UK.

The main strength of this study, setting it apart from most literature on this topic, is that the data were derived from a real-life situation where GP have been employing routine FIT as an adjunct to diagnosis in all patients presenting with lower GI symptoms, including rectal bleeding. In addition, linkage to the SCR provides assurance that a subsequent diagnosis of CRC in a patient with f-Hb <20 µg/g but not initially referred was extremely rare.

The main weakness is that only CRC was considered and data on other important conditions, especially higher risk adenomas and inflammatory bowel disease, are not presented. This is because colonoscopy data would have been necessary for these data to have been accurately assessed, and only 53.1% of patients with a f-Hb result were referred for investigation. In addition, the focus of waiting time targets for symptomatic patients is focused on CRC detection. Moreover, since different FIT systems do not give the same numerical estimates of f-Hb,^37^ the numerical data here may not be transferrable across systems.

## Conclusions

Current evidence clearly points to moving away from the assessment of specific presenting symptoms to determine a patient’s CRC risk towards a FIT-based algorithm with symptoms as the entry point. The work presented here, building on a recent study done in three NHS Boards in Scotland^38^ and performed according to the STARD (Standards For Reporting Diagnostic Accuracy Studies) guidelines on assessing diagnostic accuracy,^39^ and in our previous publication,^14^ provides compelling evidence that FIT can be employed safely in primary care. In addition, the detailed presentation of the effect of a range of f-Hb thresholds provides a guide as to how FIT can best be used, and sets out the implications of varying the f-Hb threshold used to determine the need to refer a patient for investigation of lower GI symptoms.
